# Portable Colorimetric Hydrogel Test Kits and On-Mobile Digital Image Colorimetry for On-Site Determination of Nutrients in Water

**DOI:** 10.3390/molecules27217287

**Published:** 2022-10-26

**Authors:** Worawit Wongniramaikul, Bussakorn Kleangklao, Chanita Boonkanon, Tarawee Taweekarn, Kharittha Phatthanawiwat, Wilasinee Sriprom, Wadcharawadee Limsakul, Wanchitra Towanlong, Danai Tipmanee, Aree Choodum

**Affiliations:** Integrated Science and Technology Research Center, Faculty of Technology and Environment, Prince of Songkla University, Phuket Campus, Kathu, Phuket 83120, Thailand

**Keywords:** nutrient, hydrogel, digital image colorimetry, test kit, on-site determination

## Abstract

Portable colorimetric hydrogel test kits are newly developed for the on-site detection of nitrite, nitrate, and phosphate in water. Griess-doped hydrogel was prepared at the bottom of a 1.5 mL plastic tube for nitrite detection, a nitrate reduction film based on zinc powder was placed on the inner lid of a second 1.5 mL plastic tube for use in conjunction with the Griess-doped hydrogel for nitrate detection, and a molybdenum blue-based reagent was entrapped within a poly(vinyl alcohol) hydrogel matrix placed at the bottom of a third 1.5 mL plastic tube to detect phosphate. These test kits are usable with on-mobile digital image colorimetry (DIC) for the on-site determination of nutrients with good analytical performance. The detection limits were 0.02, 0.04, and 0.14 mg L^−1^ for nitrite, nitrate, and phosphate, respectively, with good accuracy (<4.8% relative error) and precision (<1.85% relative standard deviation). These test kits and on-mobile DIC were used for the on-site determination of nutrients in the Pak Bang and Bang Yai canals, the main canals in Phuket, Thailand. The concentrations of nitrite, nitrate, and phosphate were undetectable to 0.60 mg L^−1^, undetectable to 2.98 mg L^−1^, and undetectable to 0.52 mg L^−1^, respectively.

## 1. Introduction

Water pollution is a major challenge that humanity is facing. Specifically, nutrient pollution caused by excess nitrogen and phosphorus in the water can have diverse, extensive effects on public health, environments, and economies. Nitrogen and phosphorus are the most important and abundant nutrients that occur naturally in aquatic ecosystems. However, substances from human activities, such as fertilizers, wastewater, automobile exhaust, and animal waste, introduce excess amounts of these nutrients into the ecosystem faster than it can adapt. This results in their overabundance, leading to increased primary production of biomass, oxygen depletion, and toxic algal blooms (eutrophication) [1]. Eutrophication is a serious environmental problem receiving substantial attention from the scientific community. It can kill mollusks, fish, and other inhabitants of aquatic ecosystems [1,2,3]. Algal growth in streams and rivers can block water pipelines and turbines [2,3]; moreover, the presence of toxic red algae and dinoflagellates on seashores can cause income loss and economic disruptions [2,3] and affect human health [2].

Nitrite (NO_2_^−^) and nitrate (NO_3_^−^) are nitrogen nutrients that are integral parts of the nitrogen cycle in the environment, whereas phosphates are the most common phosphorus nutrients. Nitrite is an important indicator of water quality, and it is generally found in surface water at low concentrations (0.07 mg L^−1^ NO_2_^−^–N) because it oxidizes to nitrate easily. Orthophosphates are the most stable phosphates, and they can exist in a variety of species, such as orthophosphate/phosphate (PO_4_^3−^), hydrogen phosphate (HPO_4_^2−^), dihydrogen phosphate (H_2_PO_4_^−^), and phosphoric acid (H_3_PO_4_), based on different pH values [4]. Phosphate at concentrations of 25 μg L^−1^ to 0.03 mg L^−1^ [1] or above 0.02 mg L^−1^ [5] can cause eutrophication. According to the World Health Organization (WHO) standards, the permissible limits of nitrate, nitrite, and phosphate in surface water are 50, 3, and 5 mg L^−1^, respectively [6].

Numerous instrumental analysis methods detect nitrate, nitrite, and phosphate in water [7,8,9,10]. The colorimetric method is a simple, widely adopted approach where the Griess reaction is used to detect nitrate and nitrite [8], and a molybdenum blue-based reaction is applied for phosphate [10]. The Griess reaction is based on the diazotization of aromatic amines, such as sulfanilamide, using acidified nitrite, forming a diazonium cation. This cation is further reacted with n-(1-naphthyl)-ethylenediamine dihydrochloride (NED) by a coupling reaction to form a pink azo dye [7,11,12]. Nitrate is detected by reducing it to nitrite and then conducting the Griess reaction. Various reductants have been reported, such as titanium chloride, hydrazine, cadmium [13], and zinc powder, an environmentally friendly, cost-effective reductant [7,11]. For the molybdenum blue-based method for phosphate detection, ammonium molybdate reacts with orthophosphate under strong acidic conditions to form a Keggin ion and then is reduced by ascorbic acid to generate a molybdenum blue complex [14,15]. Potassium antimony tartrate is commonly used as a source of antimony to increase the reaction rate and eliminate the need for a heating process to form a stable molybdenum blue product [15,16]. Polymeric test kits involving the use of polymers to stabilize and/or support colorimetric reagents have also been reported for the detection of these nutrients [11,17,18,19,20]. They are better than conventional liquid test kits because they are portable and carry less risk from hazardous chemicals.

In this paper, hydrogel test kits were developed for simple, rapid on-site quantitative analysis of nitrite, nitrate, and phosphate in water samples for the first time. Microliter-scale (<75 µL) Griess and molybdenum blue-based reagents were entrapped within a poly(vinyl alcohol) (PVA) matrix to fabricate small, portable test kits in 1.5 mL plastic tubes for nitrite and phosphate, respectively. For nitrate detection, zinc powder was entrapped within a tapioca starch film to fabricate a nitrate reduction film on the inner lid of the 1.5 mL plastic tube containing the Griess-doped PVA nitrite test kit. This film reduces nitrate to nitrite, which is then subjected to a colorimetric reaction with the Griess-doped PVA nitrite test kit at the bottom of the tube. These test kits will make the colorimetric testing of these nutrients easier and more portable while eliminating any risk from hazardous chemicals. They are also environmentally friendly methods since PVA and tapioca starch are biodegradable polymers. Digital image colorimetry (DIC) based on a smartphone application (on-mobile DIC) was used in conjunction with the developed hydrogel test kits to eliminate the need for a spectrophotometer [8,9]. This enabled the developed method for rapid on-site quantitative analysis of nutrients in water samples.

## 2. Results and Discussion

### 2.1. Preparation of In-Tube Hydrogel Test Kits for Nutrient Detection

**Figure 1:**
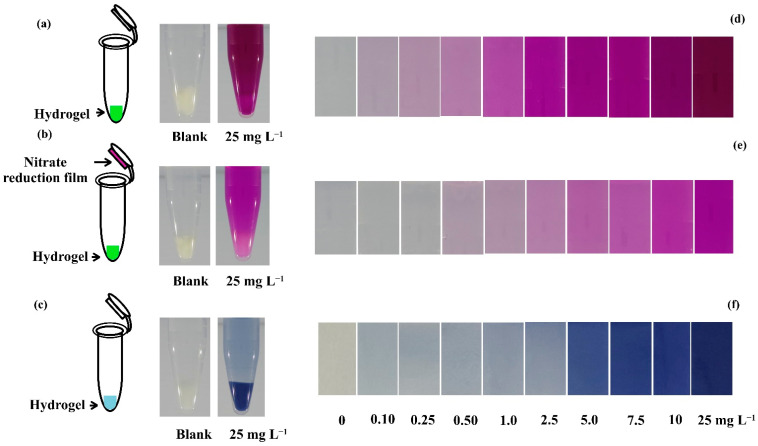
In-tube hydrogel test kits for (**a**) nitrite, (**b**) nitrate, and (**c**) phosphate detection and the colorimetric products obtained from the testing of standard solutions with the (**d**) nitrite, (**e**) nitrate, and (**f**) phosphate test kits.

The in-tube hydrogel test kits for nitrite (Figure 1a) and nitrate (Figure 1b) were synthesized by modifying a previously reported procedure [11]. The optimal ingredient in the nitrite test kit was the polymer mixture, containing 1 mL PVA solution, 0.75 mL Griess reagent, and 5 μL poly(ethylene glycol) diglycidyl ether (EGDE) [11]. A smaller amount of this polymer mixture (100 µL) relative to that in [11] (400 µL) was transferred into a 1.5 mL plastic tube and frozen instead of being placed in a 24-well plate. Twenty-four test kits could be synthesized in a 24-well plate in [11], but a reflection from nearby test kits may affect the quantification. The use of individual containers, namely plastic tubes, prevented such interference and any potential cross-contamination during testing. For nitrate detection, the abovementioned nitrite test kit was used in conjunction with the nitrate reduction film in [11]. The tube for nitrate testing thus had the nitrite-testing hydrogel at the bottom and a nitrate reduction film on its inner lid.

**Figure 2:**
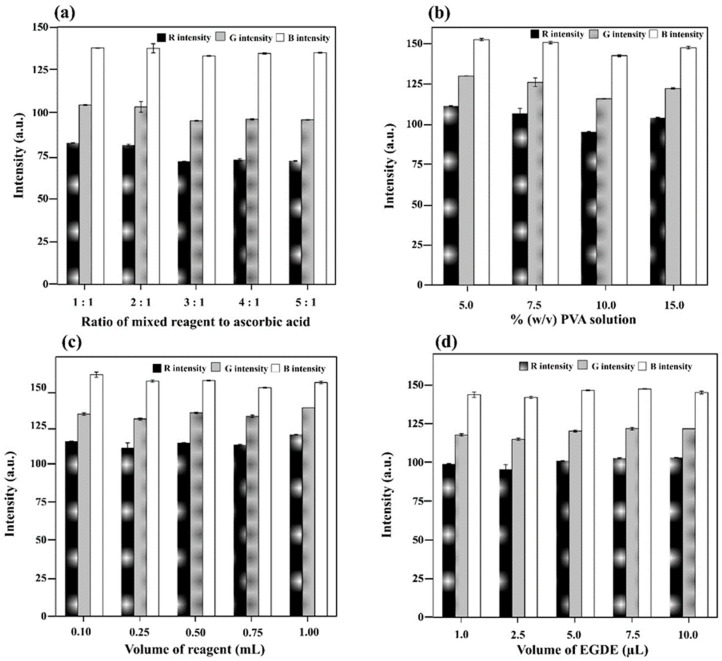
Influence of colorimetric ingredients: (**a**) ratio of the reagent mixture and ascorbic acid in the colorimetric reagent, (**b**) % *w/v* of the PVA solution, (**c**) volume of the colorimetric reagent in the polymer mixture, and (**d**) volume of EGDE on the RGB intensity of the colorimetric product obtained from the testing of the standard phosphate solution at 5 mg L^−1^.

For the in-tube hydrogel test kit for phosphate (Figure 2c), the ingredients in a previous study [19] were initially used for re-optimization in which a 1.5 mL plastic tube was used instead of a 200 µL plastic tube [19]. This can facilitate on-site testing, where smaller test kits may be unsuitable. The optimal ratio of the reagent mixture (4 mL 10% *w/v* ammonium molybdate, 10 mL 4.5 M sulfuric acid, and 1 mL 1.25% *w/v* potassium antimony tartrate) and the 20% ascorbic solution was determined. The findings showed that 3:1 provided the darkest color product with the lowest RGB intensities (I_R_, I_G_, and I_B_; Figure 2a). The molybdenum blue-based reagent was thus prepared by mixing 4 mL 10% *w/v* ammonium molybdate, 10 mL 4.5 M sulfuric acid, and 1 mL 1.25% *w/v* potassium antimony tartrate with 3 mL 20% ascorbic acid solution. The concentration of the PVA solution (% *w*/*v*) was also optimized from 5% to 15%, and a 10% *w/v* PVA solution provided the darkest color product (lowest I_R_, I_G_, and I_B_; Figure 2b) with good physical characteristics of the hydrogel. The volume of the molybdenum blue-based reagent in 10% *w/v* PVA solution (1 mL) was then optimized, and results indicated that 0.25 mL provided the darkest color product with good characteristics (Figure 2c). Thus, 1 mL 10% *w/v* PVA solution was mixed with 0.25 mL molybdenum blue-based reagent to prepare the polymer mixture for phosphate detection. The amount of the cross-linker (EGDE) was also optimized by adding it (1 to 10 µL) into the obtained polymer mixture (1.25 mL), and 2.5 µL was selected as the optimal value because it yielded the darkest color product and good hydrogel characteristics (Figure 2d). The mixture (100 µL) was transferred into the bottom of a 1.5 mL plastic tube and then frozen.

**Scheme 1:**
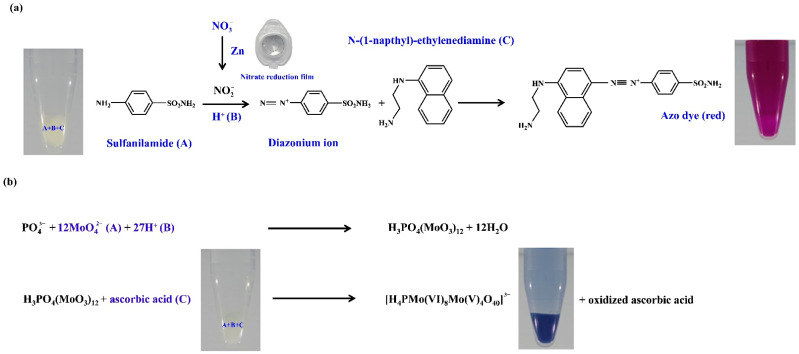
The detection mechanism of (**a**) nitrate and nitrite, and (**b**) phosphate using hydrogel colorimetric test kits.

Thus, the proposed in-tube hydrogel test kits for nitrite, nitrate, and phosphate were obtained from a combination of physical cross-linking (cryogenic treatment) and chemical cross-linking (use of EGDE as a cross-linker), as done in our previous studies [11,19] but modified to facilitate on-site detection. The detection mechanism of nutrients is shown in Scheme 1.

### 2.2. Characterization of Hydrogel Test Kits

**Figure 3:**
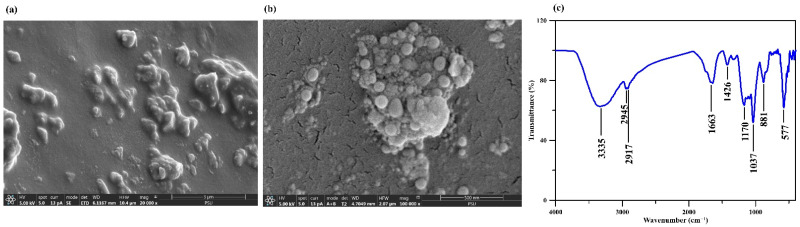
SEM images of the hydrogel used for phosphate detection at (**a**) 20,000× and (**b**) 100,000× and (**c**) its FTIR spectrum.

The characterization of the hydrogel test kit for phosphate was investigated. The scanning electron microscopy (SEM) images of the test kit revealed a homogeneous entrapment of the molybdenum blue-based reagent within the hydrogel matrix (Figure 3a,b). The presence of the molybdenum blue-based reagent in the hydrogel test kit was confirmed by the Fourier-transform infrared (FTIR) spectrum (Figure 3c). A band with the maximum absorption at 3335 cm^−1^ was attributed to the O–H stretching from sulfuric acid and/or ascorbic acid; this may have overlapped with the O–H stretching from the PVA, which appeared as a broad absorption peak at 3268 cm^−1^ in the blank hydrogel (PVA and EGDE without reagent) [11]. It may have also overlapped with the N–H stretching vibration from ammonium molybdate, which commonly presents at 3156 to 3163 cm^−1^ [21]. A stretching vibration of the alkyl groups (R–CH2) from EGDE and/or PVA was observed at a peak between 2945 and 2917 cm^−1^, a slight shift from that of the blank hydrogel (2939 and 2910 cm^−1^ [11]). The stretching of Mo–O–Mo bonding from ammonium molybdate [21] was observed at 881 cm^−1^, confirming the presence of ammonium molybdate in the test kit. The bending of Mo–O–Mo bonding from ammonium molybdate, which commonly presents at 990 cm^−1^ [21], may have overlapped with the vibration of the C–O bonding from potassium antimony tartrate (1150 to 1085 cm^−1^) [22] and/or C–O–C stretching vibration from the cross-linking of PVA and EGDE (1089 cm^−1^ [11]) and thus appeared at 1037 cm^−1^. The peak at 1676 cm^−1^ was assigned to the C=C stretching from ascorbic acid [23,24], whereas the peak at 1331 cm^−1^ might be attributed to the vibration of enol hydroxy [24]. The peaks at 762 to 669 cm^−1^ might be attributed to the Sb–O from potassium antimony tartrate [22].

### 2.3. In-Tube Colorimetric Testing of Nutrients by Hydrogel Test Kits

Pink-violet products were obtained from nitrite and nitrate testing, whereas blue products were obtained from phosphate testing (Figure 1). They were based on the reaction between the nutrient ions in the sample/standard solutions and the appropriate reagents entrapped in the hydrogel. Nitrite ions reacted with the acidic sulfanilamide, forming diazonium cations, which further reacted with NED to form azo dye [11]. Nitrate ions were reduced to nitrite by the zinc powder entrapped within the film, and the resulting nitrite reacted in the same manner as the above nitrite ions. During phosphate testing, phosphate ions reacted with the ammonium molybdate in the aqueous sulfuric acid medium, forming 12-molybdophosphoric acid that further reduced via ascorbic acid to form phosphomolybdate blue [10,19,25]. Potassium antimony tartrate was used to catalyze the reaction and inhibit the formation of a common interference (silicomolybdic acid).

**Figure 4:**
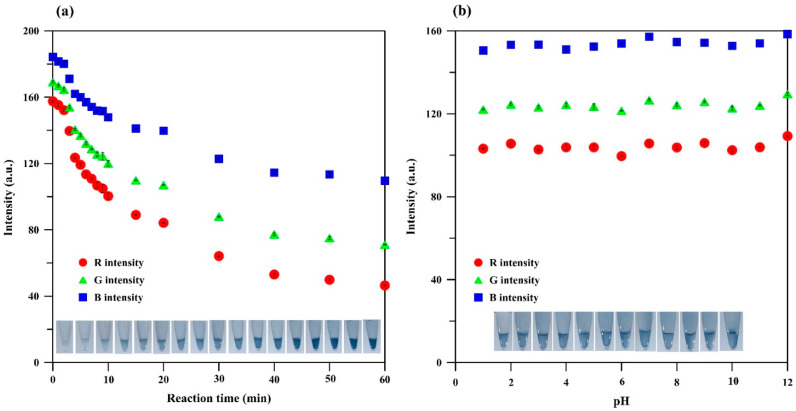
Influence of the (**a**) reaction time and (**b**) pH.

The blue products darkened with increasing reaction time (Figure 4a), and 10 min was selected for quantitative analysis. This was faster than that in a previous report (30 min) [26] and that of a lab-on-chip analyzer (35 min) [25] but longer than that in the Environmental Protection Agency (EPA) 365.3 standard method (5 min) [27]; this difference may be attributable to the hydrogel matrix. Furthermore, a pink-violet product was observed immediately after nitrite testing, and the test required 3 min to reach equilibrium [11], which was faster than that in the EPA 1686 standard method (10 min) [28] and that in a previous study (20 min) [29]. As for nitrate testing, 4 min was needed to reduce nitrate to nitrite before testing [11]. Although the hydrogel matrix may have affected the reaction time of the phosphate test, it can stabilize molybdenum blue-based reagents for at least a year with changes in the red, green, and blue (RGB) intensities (I_R_, I_G_, and I_B_) of the blue products (2.5 mg L^−1^) for −3.4%, −2.3%, and +0.9%, respectively. Moreover, the hydrogel test kit for nitrite can be frozen (−20 °C) for three months, and the nitrate reduction films can be stored in a desiccator (25 °C) for six months [11]. Therefore, these hydrogel test kits can reduce complications and time consumption for fresh reagent preparation before on-site detection. The small plastic tubes are also more portable, as they do not require carrying the required reagents in their liquid form.

The influence of pH on the colorimetric testing of the hydrogel test kit for phosphate was investigated by adjusting the standard solution of phosphate (5 mg L^−1^) to pH 1 to 12 using HCl and NaOH before testing. The results (Figure 4b) revealed that pH did not affect the intensity of the reflected light from the digital image of the blue product. Therefore, the test kit can be used for phosphate detection without pH adjustment and is suitable for on-site determination. The hydrogel test kits for nitrite and nitrate showed good results at pH values of 6 to 8 [11], which are within the range recommended by the EPA standard method (pH 5 to 9) and in the range of the common pH of seawater (pH 6 to 7). Therefore, the in-tube colorimetric test of nutrients was completed using the developed hydrogel test kits without any pH adjustment for both the establishment of calibration curves and the analysis of real samples.

Darker products were obtained when the nutrient concentrations were increased (Figure 1d–f). The pink-violet products from nitrate were paler than those from nitrite at the same concentration; this may contribute to nitrate reduction efficiency, as discussed and observed in many reports [29,30,31]. Analysis of RGB intensities from the digital images of these products can be used for quantitative analysis.

### 2.4. Nutrient Quantification via On-Mobile DIC

**Figure 5:**
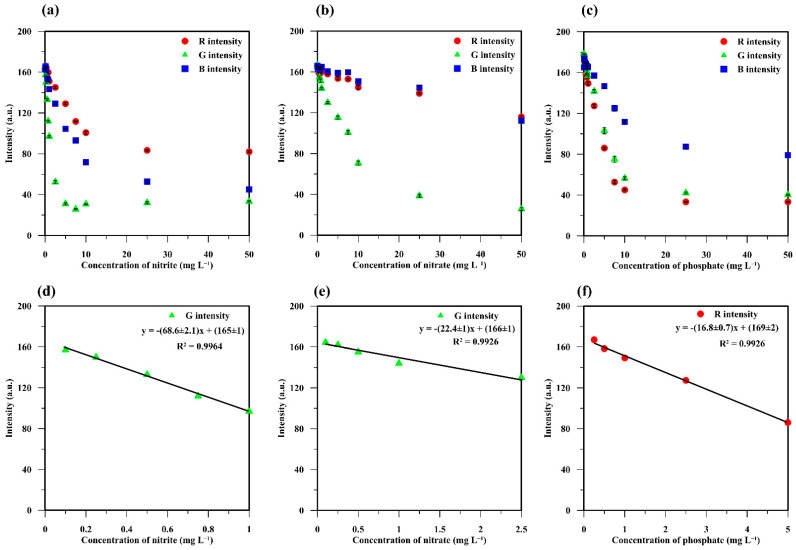
Relationships between the RGB intensities and concentrations of (**a**) nitrite, (**b**) nitrate, and (**c**) phosphate and their linear relationships, which can be used for quantitative analysis of (**d**) nitrite, (**e**) nitrate, and (**f**) phosphate.

An on-mobile DIC system consisting of a custom-built RGB analysis box and a custom-built RGB analysis smartphone application was used for on-site quantitative analysis of nutrients in water samples without any effect from environmental light [11]. The RGB values (I_R_, I_G_, and I_B_) were obtained from the analysis of the color products (Figure 1d–f) using the custom-built color analysis application. These RGB values were related to the intensities of the light reflected from the color products, which were negatively correlated to the nutrient concentrations (Figure 5a–c).

The intensity of the reflected green light from the pink-violet products from nitrite (Figure 5a) and nitrate (Figure 5b) was lower than those of blue (I_B_) and red (I_R_), which agrees with a previous report [11]. This was because of the higher absorption of green light (490 to 590 nm) of these products, as reported in the literature (539 nm [11], 540 nm [8], 543 nm [32], and 548 nm [20]. For the blue products, I_B_ was higher than I_G_ and I_R_ because of the higher reflection of blue light (Figure 5c). Such blue products have been found to provide the maximum light absorption at wavelengths of 630 nm [33], 650 nm [27], 700 nm [25], and 850 nm [26], which correspond with the highest absorption of red light (580 to 700 nm) [33,34,35], thus providing the lowest reflected value of I_R_, as shown in Figure 5c. A portion of the relationships between the RGB values and the nutrient concentrations were fundamentally linear and can thus be applied to the quantification of these nutrients in real samples.

### 2.5. Analytical Performance and Method Validation

The analytical performance of the in-tube hydrogel test kits and the on-mobile DIC system developed for nutrient analysis is summarized in Table 1. I_G_ exhibited a linear relationship with nitrate and nitrite concentrations in the range of 0.1 to 1 mg L^−1^ (Figure 5d,e), with a good correlation coefficient (R = 0.9963 and 0.9982, respectively). The obtained limit of detection (LOD) (0.02 mg L^−1^) and limit of quantification (LOQ) (0.07 mg L^−1^) for nitrite analysis were lower than those of nitrate (0.04 and 0.14 mg L^−1^, respectively). These are lower than the concentration limits in surface water recommended by the WHO of 3 and 50 mg L^−1^ for nitrite and nitrate, respectively [6,36]. The LOD and LOQ for phosphate analysis were 0.14 and 0.47 mg L^−1^, equivalent to 0.05 and 0.15 mg PO_4_–P L^−1^, respectively. This LOD is lower than the maximum concentration of total phosphorus in discharge from municipal wastewater treatment plants in Thailand (2 mg L^−1^ [37], recommended total PO_4_–P in flowing water by the US EPA (≤0.1 mg L^−1^) [26,38] and maximum allowable concentration of phosphate in surface water recommended by the WHO (5 mg L^−1^, [6]). It is equal to the US EPA recommendation for total PO_4_–P in streams at points where they discharge into lakes or reservoirs (≤0.05 mg L^−1^) [26,38]. The percentage relative error (% RE) obtained from analyzing a standard solution of phosphate (0.75 mg L^−1^) against the concentration quantified by the I_R_ calibration graph was +3.0%, whereas +3.0% and −4.8% RE were obtained from the I_G_ calibration graphs for nitrite and nitrate, respectively. These results indicate the excellent accuracy of the developed method. A good intraday precision (0.70% to 1.52% relative standard deviation (RSD)) was also obtained from testing 12 hydrogel test kits for phosphate, whereas the interday precision from nine test kits over three days was in the range of 0.70% to 1.69% RSD. The performance of the developed method was similar to that in other reports (Table 2), but it has the advantage of enabling on-site quantification without the need for expensive instruments.

### 2.6. Influence of Potential Interferences on Nutrient Quantification

**Figure 6:**
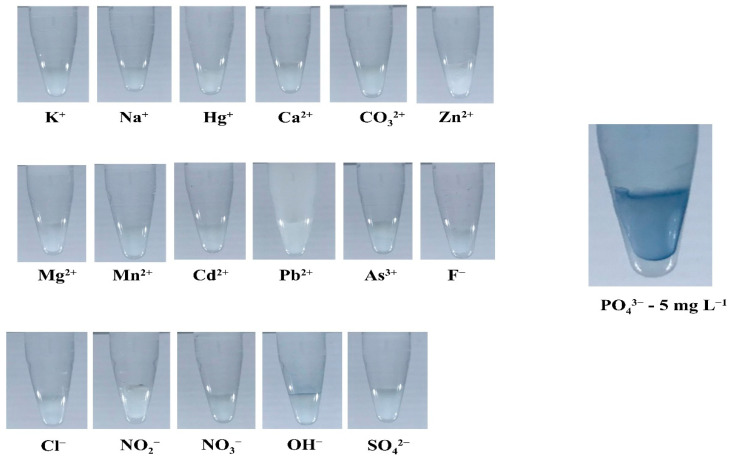
Colorimetric products obtained from the testing of various interference ions with the hydrogel test kit for phosphate.

Various ions at ~10,000 mg L^−1^ were tested with the hydrogel test kits for phosphate. No color product was observed from any of the ions (Figure 6), indicating its excellent selectivity. Each ion was then added to standard phosphate solutions (5 mg L^−1^) to investigate the tolerance ratios. The results in Table 3 show that phosphate can tolerate these interfering ions, even at high concentrations. The nitrate and nitrite test kits can also tolerate interfering ions, even at high concentrations, as discussed in [11]. Likewise, the influence of the nitrate ions on nitrite quantification, the influence of the nitrate reduction film on nitrite quantification, the influence of nitrite ions on nitrite quantification, and the influence of seawater had little effect on the quantification accuracy of nitrate and nitrite [11]. The influence of a seawater matrix on phosphate quantification was also evaluated by preparing standard phosphate solutions at various concentrations (0 to 5 mg L^−1^) using seawater sampled from the Andaman Sea (far from human activity) instead of ultrapure water. The sensitivity of the I_R_ standard graph from seawater was different from that obtained using ultrapure water by −3.83%, indicating that the seawater matrix exerted little effect. Ascorbic acid and formic acid were also tested with nitrate, nitrite, and phosphate test kits, and no color products were observed. Phosphate (5 mg L^−1^) can tolerate ascorbic acid and formic acid at high concentrations (10,000 mg L^−1^; Table 3). Nitrate and nitrite (5 mg L^−1^) can tolerate formic acid at 10,000 mg L^−1^, but at lower concentrations for ascorbic acid (25 mg L^−1^).

### 2.7. Nutrient Quantification in Surface Water in Phuket

The developed in-tube hydrogel test kits and the on-mobile DIC system were used to determine nutrients in surface water sampled from the Pak Bang canal, Bang Yai canal, and seawater at the estuaries of both canals. The concentrations of nitrite, nitrate, and phosphate ranged from undetectable to 0.60 mg L^−1^, undetectable to 2.98 mg L^−1^, and undetectable to 0.52 mg L^−1^, respectively (Table 4). The quantified concentrations obtained from the developed method showed strong, positive correlations with the standard method (spectrophotometry), with a Pearson coefficient of +0.983. They showed no significant differences from those obtained using standard methods in the independent-samples t-test (P = 0.592, t = 0.537, n = 288). The percentage recovery was also investigated using a spiked standard solution with the real samples, and the percentage recoveries were 90.5 ± 0.9 to 108.9 ± 0.4 (Table 5).

The nitrate concentration was commonly higher than that of nitrite; this may have been due to the oxidation of nitrite to nitrate. It was also higher than that of phosphate, as in other reports [45,46], and this may have been because nitrate leaches more easily than other contaminants in some soil types [45]. All of them did not exceed the limit set by the WHO for surface water (50, 3, and 5 mg L^−1^ for nitrate, nitrite, and phosphate, respectively [6,36]. The highest nutrient concentrations were sampling points P10 to P15 in Pak Bang canal; this may be attributable to discharge from the Patong municipal wastewater treatment plant, which is very close to P14. Phosphate was found by the standard method at these sampling points in the range of 0.30 to 0.50 mg L^−1^ (mean: 0.37 ± 0.07 mg L^−1^, median: 0.39 mg L^−1^, n = 17), whereas a mean phosphate concentration of 0.50 ± 0.03 mg L^−1^ (median: 0.50 mg L^−1^, n = 2) was found by the developed method at sampling points P12 and P14. The higher mean concentration of the developed method was due to its LOQ of 0.47 mg L^−1^, which means that the method cannot quantify phosphate in the range of the standard method (0.30 to 0.42 mg L^−1^). The nutrient pollution index (*NPI*) was calculated by the sum of the nitrate, nitrite, and phosphate indices [46,47].

(1) $$NPI=\frac{C_{nitrate}}{MAC_{nitrate}}+\frac{C_{nitrite}}{MAC_{nitrite}}+\frac{C_{phosphate}}{MAC_{phosphate}}$$

where *C_i_* is the mean concentration of nutrients in water and *MAC_i_* is the maximum allowable concentration of nutrients in surface water recommended by the WHO (50, 3, and 5 mg L^−1^ for nitrate, nitrite, and phosphate, respectively [6,36]. Pollution was categorized as absent (*NPI* < 1), moderate (1 ≤ *NPI* ≤ 3), considerable (3 < *NPI* ≤ 6), or very high (*NPI* > 6) [46]. The *NPI* of the Bang Yai canal (0.21) was lower than that of the Pak Bang canal (0.25), but both values indicated an absence of pollution, similar to the *NPI* of 0.27 at sampling points P10 to P15.

## 3. Materials and Methods

### 3.1. Materials

Sodium nitrate, sodium nitrite, ammonium heptamolybdate tetrahydrate, and potassium antimony tartrate were purchased from Carlo Erba (Milan, Italy). Disodium hydrogen phosphate and phosphoric acid (85%) were from Ajax Finechem (Sydney, Australia), and sulfuric acid was obtained from J.T. Baker (Radnor, PA, USA). NED and ascorbic acid were bought from Fisher Scientific (Leicestershire, UK), and sulfanilamide was purchased from Panreac (Barcelona, Spain). PVA (Mw = 85,000 to 124,000 g mol^−1^, >99% hydrolyzed), zinc powder, and EGDE (98%) were from Sigma-Aldrich (Saint Louis, MO, USA). Tapioca starch (Jaydee Brand, Nakhon Pathom, Thailand) was bought from a supermarket; a spectrophotometer showed that it had 27% amylose content, which accorded with the Thai agricultural standard TAS 4000-2003 (in-house method TE-PH-021, based on quality and testing of Thai Hom Mali rice, 2004, Department of Agriculture, Bangkok, Thailand). All standard solutions were prepared with ultrapure water from a water purification system (Merck, Darmstadt, Germany).

### 3.2. Preparation of Hydrogel Test Kits and Nitrate Reduction Film

The hydrogel test kits for nitrite, nitrate, and phosphate detection were developed by entrapping Griess and molybdenum blue-based reagents within PVA hydrogel matrices. Each PVA solution was prepared by dissolving PVA granules (1 g) in ultrapure water (10 mL) at 120 °C and stirring the mixture for 30 min. The clear, viscous solution was cooled at room temperature before adding either the Griess or molybdenum blue-based reagent, and then a cross-linker (EGDE). The Griess reagent was prepared by dissolving 0.3 g sulfanilamide, 45 mg NED, and 1.5 mL 85% phosphoric acid in 10 mL ultrapure water. The molybdenum blue-based reagent was prepared by mixing 4 mL 10% *w/v* ammonium molybdate, 10 mL 4.5 M sulfuric acid, and 1 mL 1.25% *w/v* potassium antimony tartrate with a 20% ascorbic solution using an appropriate ratio. The Griess reagent (0.75 mL) or molybdenum blue-based reagent (0.25 mL) was mixed with 1 mL of the PVA solution, the mixture was stirred for a few minutes, 2.5 μL EGDE was added, and the mixture was stirred for another minute. Each polymer mixture (100 μL) was transferred into the bottom of a 1.5 mL plastic tube and kept in a freezer for 24 h, and the result was the hydrogel test kits. Blank hydrogel test kits were also prepared using the same procedure but without the reagents.

The nitrate reduction film was prepared per our previous report [11]. Tapioca starch (0.3 g) was dispersed in 10 mL ultrapure water, and the mixture was heated under continuous stirring to obtain a clear, viscous solution. Zinc powder (4 mg) was added to the cooled solution and stirred for a minute. The mixture (50 μL) was then transferred to the flat cap of a 1.5 mL plastic tube and incubated at 120 °C for 15 min to fabricate the film. The lid was cooled to room temperature and then stored in a Ziploc plastic bag in a desiccator until further use.

### 3.3. Characterization of Hydrogel Test Kit for Phosphate

The morphology of the hydrogel test kit for phosphate was investigated using scanning electron microscopy (SEM; Quanta 400, FEI, Eindhoven, The Netherlands), and the functional groups were investigated using Fourier-transform infrared (FTIR) spectrophotometry (Vertex 70, Bruker, Ettlingen, Germany) with the KBr pellet method.

### 3.4. Quantitative Analysis via Colorimetric Testing and DIC

Standard solutions of sodium nitrate, sodium nitrite, and disodium hydrogen phosphate (0−50 mg L^−1^) were prepared by diluting stock solutions (500 mg L^−1^) with ultrapure water. For colorimetric testing, the standard solutions (~1.5 mL) were added to the plastic tubes containing the hydrogel test kits and left for 10 min. Three replications were conducted at each standard concentration of nitrate, nitrite, and phosphate. DIC based on a custom-built smartphone application was then applied for quantitative analysis. The colorimetric product was placed in a sample holder inside a custom-built RGB analysis box (8.5 × 9.5 × 6.25 inches) modified from previous works [11,12,48]. A Huawei P20 (12 MP backside-illuminated complementary metal oxide semiconductor (CMOS)) was then placed in a slot on the side of the box for color analysis. The custom-built smartphone application was then opened, and each test kit’s color product was imaged with the smartphone camera with controlled illumination for reproducible image analysis. A 50 × 50-pixel image patch from the image recorded from the test kit was manually selected three times; the manual selection was for the three test kits for each nutrient concentration. The average RGB intensities from these test kits (nine values in total) were used as a single data point to establish calibration curves by plotting the average I_R_, I_G_, and I_B_ values of the color products against the standard concentrations.

### 3.5. System Performance and Method Validation

System performance parameters were investigated: the linear range, correlation coefficient, sensitivity, LOD, and LOQ. The LOD and LOQ were calculated based on the International Council for Harmonisation (ICH) harmonized tripartite guideline [49], whereas precision was expressed as % RSD. Intraday precision was investigated using 12 test kits (n = 12 test kits) on the same day, while interday precision was studied for three days (n = 9 test kits). The accuracy was reported in % RE, which was evaluated by analyzing the known concentration of the standard solutions (0.75 mg L^−1^) against their established calibration curves and recovery percentage from real spiked samples.

### 3.6. Quantification of Nutrients in Surface Water

**Figure 7:**
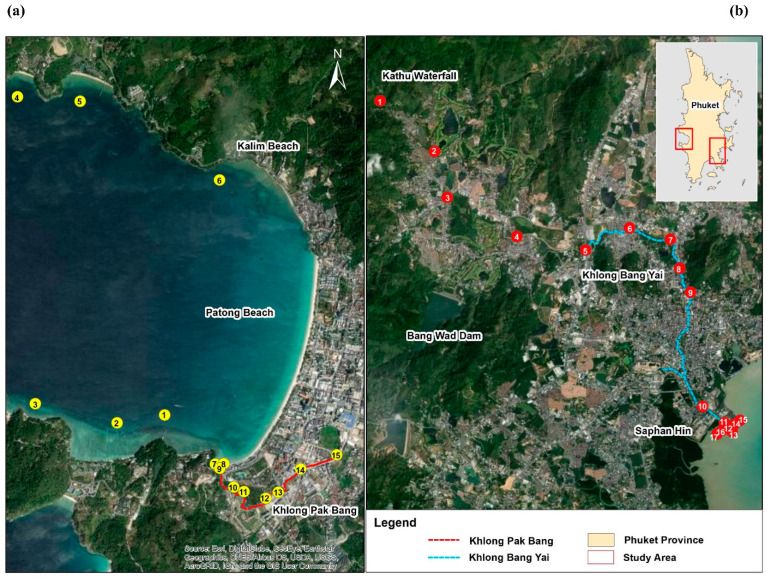
Water sampling points in Thailand: (**a**) Pak Bang canal, Patong; (**b**) Bang Yai canal, Phuket.

Surface water and seawater samples were randomly obtained from the Pak Bang canal, Bang Yai canal, and estuaries of both canals. A total of 135 grab samples were collected from 15 sampling points from the Pak Bang canal (Patong, Phuket, Thailand; Figure 7), whereas 153 samples were obtained from 17 sampling points from the Bang Yai canal, which is the main canal of Phuket. They were collected once a month for three months (September to November 2020) in polypropylene bottles with minimal air space and stored in ice in a styrene foam box. These samples were filtered through a 0.20 µm cellulose acetate membrane before being analyzed via the proposed method (use of the test kits and on-mobile DIC) and the standard method (spectrophotometry) within a few hours.

## 4. Conclusions

In-tube hydrogel test kits were used in conjunction with on-mobile DIC for on-site analysis of nutrients in surface water and seawater in Phuket, Thailand. Pink-violet products were obtained from nitrite testing using Griess-doped hydrogel test kits. A reduction film based on zinc powder was used to reduce nitrate to nitrite before testing the resultant nitrite with a Griess-doped hydrogel test kit for nitrate testing. Blue products were obtained from phosphate testing with a molybdenum blue-doped hydrogel test kit. The RGB intensities (I_R_, I_G_, and I_B_) of these colorimetric products can be analyzed using a custom-built smartphone application and used to quantify these nutrients with good analytical performance. The relationships between I_G_ and the nitrite and nitrate concentrations were linear in the range of 0.1 to 1 mg L^−1^, with a good correlation coefficient (R > 0.99), similar to that of the relationship between I_R_ and the phosphate concentration in the range of 0.25 to 5 mg L^−1^. The obtained LODs for these nutrients were lower than the maximum allowable concentration of nutrients in surface water recommended by the WHO; they were accurate (<4.8% RE) and precise (<1.85% RSD). These test kits and on-mobile DIC were then used for the on-site determination of nutrients in the Pak Bang and Bang Yai canals, which are the main canals in Phuket, Thailand. The nitrite, nitrate, and phosphate concentrations ranged from undetectable to 0.60 mg L^−1^, undetectable to 2.98 mg L^−1^, and undetectable to 0.52 mg L^−1^, respectively, which are under the limits set by the WHO standard. The NPIs of both canals indicated an absence of pollution.

## Figures and Tables

**Table 1:** Nutrient analysis performance of nutrient test kits and on-mobile DIC.

Parameter	Nitrite	Nitrate	Phosphate
	I_G_	I_G_	I_R_
Sensitivity	68.6 ± 2.1	22.4 ± 1.0	16.8 ± 0.7
Linear range (mg L^−1^)	0.1–1	0.1–1	0.25–5
Linearity (R^2^)	0.9964	0.9926	0.9937
Accuracy (% RE)	+3.0%	−4.8%	+3.0%
Intraday precision (% RSD; n = 12)	1.03%	1.00%	1.52%
Interday precision (n = 5)	1.85%	0.87%	1.69%
LOD (mg L^−1^)	0.02	0.04	0.14
LOQ (mg L^−1^)	0.07	0.14	0.47

**Table 2:** Analytical performance comparison of developed method and other analysis methods.

Analyte	Method	Linear Range (mg L^−1^)	LOD (mg L^−1^)	Accuracy (% RE)	Precision (% RSD)	On-Site Analysis	Ref
Nitrate	Vanadium(III)chloride-Griess spectrophotometry	0–5	0.03	-	0.8%	x	[31]
	A modified zinc-cadmium reduction with microplate spectrophotometric method	0.03–6.2	0.03	-	4.8%	x	[39]
	Griess–Ilosvay-based sequential injection analysis	0.6–8	0.09	-	4.76	x	[40]
	Griess-based test kit and on-mobile DIC	0.1–1	0.04	−4.8%	0.87%	√	This work
Nitrite	Griess-based capsule and DIC	0.01–0.2	0.01	11.7%	<3%	√	[41]
	Vanadium(III)chloride-Griess spectrophotometry	0–0.05	0.003	-	1.6%	x	[31]
	Griess–Ilosvay-based sequential injection analysis	0.03–1.4	0.01	-	3.39	x	[40]
	Griess-based test kit and on-mobile DIC	0.1–1	0.02	+3.0%	1.85%	√	This work
Phosphate	Molybdenum blue-based capsule and DIC	0.01–1	0.01	20.2%	<3%	√	[41]
	Molybdenum blue-based flow-injection analysis	0.2–15	0.05	6.2%	2.7%	x	[42]
	Phosphorescence of Mn-doped ZnS quantum dots combined with cerium(III)	0.8–30	0.26	-	3.1% (intraday)	x	[43]
	AuNPs-based colorimetric method	7.6–19	11.3	-	-	x	[44]
	Molybdenum blue-based test kit and on-mobile DIC	0.5–5	0.14	+3.0%	1.69%	√	This work

**Table 3:** Concentrations of various species that caused changes in the I_R_ of the blue products from phosphate (5 mg L^−1^) < 5%.

Co-Existing Species	Concentration (mg L^−1^)	%Change of I_R_
Zn^2+^	10,000	0.3
Mg^2+^	10,000	0.3
Mn^2+^	10,000	1.7
Cd^2+^	10,000	1.4
Na^+^	10,000	−0.6
CO_3_^2−^	10,000	0.8
SO_4_^2−^	10,000	0.6
NO_3_^−^	10,000	−3.3
Cl^−^	10,000	−2.5
K^+^	5000	+0.6
Ca^2+^	5000	+1.4
OH−	2500	−4.4
Pb^2+^	500	−4.4
Hg^2+^	500	−2.8
As^2+^	250	+1.4
NO_2_^−^	100	+0.6
F^−^	100	−1.1
Ascorbic acid	10,000	+1.6
Formic acid	10,000	−0.5

**Table 4:** Percentage recoveries from spiked real sample (P14), analyzed using in-tube hydrogel test kits and on-mobile DIC.

Analyte	Added (mg L^−1^)	Found (mg L^−1^)	Recovery (%)
Phosphate	0.00	0.52 ± 0.03	0
	0.50	1.04 ± 0.09	101.8 ± 6.6
	1.00	1.50 ± 0.06	98.5 ± 2.3
Nitrate	0.00	1.95 ± 0.02	0
	0.50	2.25 ± 0.03	92.9 ± 0.7
	1.00	2.67 ± 0.01	90.5 ± 0.9
Nitrite	0.00	0.41 ± 0.02	0
	0.50	1.06 ± 0.02	107.0 ± 5.7
	1.00	1.53 ± 0.02	108.9 ± 0.4

**Table 5:** Quantification of nutrients in water samples in Phuket, Thailand, using the developed in-tube hydrogel test kits and on-mobile DIC.

Analyte	Parameter	Concentration (mg L^−1^)		
		Nitrate	Nitrite	Phosphate
Bang Yai	Range (n = 51)	Undetectable *–2.44	Undetectable–0.50	Undetectable–0.47
	Median	1.13 (n = 44)	1.13 (n = 31)	0.47 (n = 1)
	Mean ± SD	1.08 ± 0.63 (n = 44)	0.29 ± 0.13 (n = 31)	0.47 ± 0.00 (n = 1)
B1	Range (n = 3)	Undetectable–0.26	Undetectable	Undetectable
	Median	0.26 (n = 1)	–	–
	Mean ± SD	0.26 ± 0.00 (n = 1)	–	–
B2-B10	Range (n = 27)	0.30–2.44	Undetectable–0.50	Undetectable
	Median	1.33 (n = 27)	0.33 (n = 23)	–
	Mean ± SD	1.28 ± 0.53 (n = 27)	0.31 ± 0.12 (n = 23)	–
B11-B13	Range (n = 9)	0.15–2.24	Undetectable–0.46	Undetectable–0.47
	Median	1.31 (n = 9)	0.26 (n = 6)	0.47 (n = 1)
	Mean ± SD	1.11 ± 0.72 (n = 9)	0.27 ± 0.13 (n = 6)	0.47 ± 0.00 (n = 1)
B14-B17	Range (n = 12)	Undetectable–0.96	Undetectable–0.12	Undetectable
	Median	0.34 (n = 7)	0.10 (n = 2)	–
	Mean ± SD	0.39 ± 0.26 (n = 7)	0.10 ± 0.04 (n = 2)	–
Pak Bang	Range (n = 18)	Undetectable–2.98	Undetectable–2.98	Undetectable–0.52
	Median	1.47 (n = 37)	0.39 (n = 21)	0.50 (n = 2)
	Mean ± SD	1.21 ± 0.91 (n = 37)	0.37 ± 0.11 (n = 21)	0.50 ± 0.03 (n = 2)
P1-P6	Range (n = 18)	Undetectable–0.37	Undetectable	Undetectable
	Median	0.24 (n = 12)	–	–
	Mean ± SD	0.25 ± 0.08 (n = 12)	–	–
P7-P9	Range (n = 9)	Undetectable–2.60	Undetectable–0.40	Undetectable
	Median	0.91 (n = 7)	0.26 (n = 3)	–
	Mean ± SD	1.17 ± 0.87 (n = 7)	0.30 ± 0.08 (n = 3)	–
P10-P15	Range (n = 18)	0.28–2.98	0.21–0.60	Undetectable–0.52
	Median	1.95 (n = 18)	0.40 (n = 18)	0.50 (n = 2)
	Mean ± SD	1.87 ± 0.62 (n = 18)	0.39 ± 0.11 (n = 18)	0.50 ± 0.03 (n = 2)

* Undetectable: concentration of nutrients may be found at level less than the LOQs.

## Data Availability

Not applicable.

## Sample Availability

Samples of the nutrient test kits are available from the authors.

## References

[1] Almanassra I.W., Kochkodan V., Subeh M., McKay G., Atieh M., Al-Ansari T. (2020). Phosphate removal from synthetic and treated sewage effluent by carbide derive carbon. J. Water Process. Eng..

[2] Almanassra I.W., McKay G., Kochkodan V., Ali Atieh M., Al-Ansari T. (2021). A state of the art review on phosphate removal from water by biochars. J. Chem. Eng..

[3] Huang H., Liu J., Zhang P., Zhang D., Gao F. (2017). Investigation on the simultaneous removal of fluoride, ammonia nitrogen and phosphate from semiconductor wastewater using chemical precipitation. J. Chem. Eng..

[4] Rout P.R., Shahid M.K., Dash R.R., Bhunia P., Liu D., Varjani S., Zhang T.C., Surampalli R.Y. (2021). Nutrient removal from domestic wastewater: A comprehensive review on conventional and advanced technologies. J. Environ. Manag..

[5] Seliem M.K., Komarneni S., Abu Khadra M.R. (2016). Phosphate removal from solution by composite of MCM-41 silica with rice husk: Kinetic and equilibrium studies. Microporous Mesoporous Mater..

[6] World Health Organization (2004). Guidelines for Drinking-Water Quality.

[7] Choodum A., Boonsamran P., NicDaeid N., Wongniramaikul W. (2015). On-site semi-quantitative analysis for ammonium nitrate detection using digital image colourimetry. Sci. Justice.

[8] Wang Q.-H., Yu L.-J., Liu Y., Lin L., Lu R.-G., Zhu J.-P., He L., Lu Z.-L. (2017). Methods for the detection and determination of nitrite and nitrate: A review. Talanta.

[9] Singh P., Singh M.K., Beg Y.R., Nishad G.R. (2019). A review on spectroscopic methods for determination of nitrite and nitrate in environmental samples. Talanta.

[10] Zhu X., Ma J. (2020). Recent advances in the determination of phosphate in environmental water samples: Insights from practical perspectives. TrAC—Trends Anal. Chem..

[11] Choodum A., Tiengtum J., Taweekarn T., Wongniramaikul W. (2020). Convenient environmentally friendly on-site quantitative analysis of nitrite and nitrate in seawater based on polymeric test kits and smartphone application. Spectrochim. Acta A Mol. Biomol. Spectrosc..

[12] Taweekarn T., Wongniramaikul W., Limsakul W., Sriprom W., Phawachalotorn C., Choodum A. (2020). A novel colorimetric sensor based on modified mesoporous silica nanoparticles for rapid on-site detection of nitrite. Microchim. Acta.

[13] Pistón M., Mollo A., Knochen M. (2011). A simple automated method for the determination of nitrate and nitrite in infant formula and milk powder using sequential injection analysis. J. Autom. Meth. Manag. Chem..

[14] Nagul E.A., McKelvie I.D., Worsfold P., Kolev S.D. (2015). The molybdenum blue reaction for the determination of orthophosphate revisited: Opening the black box. Anal. Chim. Acta.

[15] Heidari-Bafroui H., Charbaji A., Anagnostopoulos C., Faghri M. (2021). A Colorimetric Dip Strip Assay for Detection of Low Concentrations of Phosphate in Seawater. Sensors.

[16] Ibnul N.K., Tripp C.P. (2021). A solventless method for detecting trace level phosphate and arsenate in water using a transparent membrane and visible spectroscopy. Talanta.

[17] Yang S., Wo Y., Meyerhoff M.E. (2014). Polymeric optical sensors for selective and sensitive nitrite detection using cobalt(III) corrole and rhodium(III) porphyrin as ionophores. Anal. Chim. Acta.

[18] Amanulla B., Palanisamy S., Chen S.-M., Chiu T.-W., Velusamy V., Hall J.M., Chen T.-W., Ramaraj S.K. (2017). Selective Colorimetric Detection of Nitrite in Water using Chitosan Stabilized Gold Nanoparticles Decorated Reduced Graphene oxide. Sci. Rep..

[19] Wongniramaikul W., Choodum A. (2017). Synthesis of Polymer Sensor for Detection of Phosphate in Water. Int. J. Chem. Eng. Appl..

[20] Nam J., Jung I.-B., Kim B., Lee S.-M., Kim S.-E., Lee K.-N., Shin D.-S. (2018). A colorimetric hydrogel biosensor for rapid detection of nitrite ions. Sens. Actuators B Chem..

[21] Sadighi S., Targhi S.K.M. (2017). Preparation of Biofuel from Palm Oil Catalyzed by Ammonium Molybdate in Homogeneous Phase. Bull. Chem. React. Eng. Catal..

[22] Barbosa V.T., de Menezes J.B., Santos J.C.C., de Assis Bastos M.L., de Araújo-Júnior J.X., do Nascimento T.G., Basílio-Júnior I.D., Grillo L.A.M., Dornelas C.B. (2019). Characterization and Stability of the Antimony-Quercetin Complex. Adv. Pharm. Bull..

[23] Bunaciu A.A., Bacalum E., Aboul-Enein H.Y., Elena Udristioiu G., Fleschin Ş. (2009). FT-IR Spectrophotometric Analysis of Ascorbic Acid and Biotin and their Pharmaceutical Formulations. Anal. Lett..

[24] Umer A., Naveed S., Ramzan N., Rafique M.S., Imran M. (2014). A green method for the synthesis of Copper Nanoparticles using L-ascorbic acid. Matéria.

[25] Clinton-Bailey G., Grand M., Beaton A., Nightingale A., Owsianka D., Slavik G., Cardwell C., Connelly D., Mowlem M. (2017). A Lab-on-Chip Analyzer for in Situ Measurement of Soluble Reactive Phosphate: Improved Phosphate Blue Assay and Application to Fluvial Monitoring. Environ. Sci. Technol..

[26] Habibah N., Dhyanaputri I.G.A.S., Karta I.W., Sundari C.D.W.H., Hadi M.C. (2018). A simple spectrophotometric method for the quantitative analysis of phosphate in the water samples. J. Sains Teknol..

[27] (1978). Phosphorous, All Forms (Colorimetric, Ascorbic Acid, Two Reagent).

[28] (2001). Nitrate/Nitrite-N in Water and Biosolids by Manual Colorimetry.

[29] García-Robledo E., Corzo A., Papaspyrou S. (2014). A fast and direct spectrophotometric method for the sequential determination of nitrate and nitrite at low concentrations in small volumes. Mar. Chem..

[30] Miranda K.M., Espey M.G., Wink D.A. (2001). A rapid, simple spectrophotometric method for simultaneous detection of nitrate and nitrite. Nitric Oxide.

[31] Schnetger B., Lehners C. (2014). Determination of nitrate plus nitrite in small volume marine water samples using vanadium(III)chloride as a reduction agent. Mar. Chem..

[32] Pasquali C.E.L., Gallego-Picó A., Hernando P.F., Velasco M., Alegría J.S.D. (2010). Two rapid and sensitive automated methods for the determination of nitrite and nitrate in soil samples. Microchem. J..

[33] Knochen M., Rodríguez-Silva J.C., Silva-Silva J. (2020). Exploitation of reaction mechanisms for sensitivity enhancement in the determination of phosphorus by sequential injection analysis. Talanta.

[34] Choodum A., Kanatharana P., Wongniramaikul W., Nic Daeid N. (2013). Using the iPhone as a device for a rapid quantitative analysis of trinitrotoluene in soil. Talanta.

[35] Choodum A., Kanatharana P., Wongniramaikul W., NicDaeid N. (2015). A sol–gel colorimetric sensor for methamphetamine detection. Sens. Actuators B Chem..

[36] World Health Organization (2003). Nitrate and Nitrite in Drinking-Water: Background Document for Development of WHO Guidelines for Drinking-Water Quality.

[37] The Ministry of Natural Resources and Environment of Thailand (2010). Notification of the Ministry of Natural Resources and Environment for Standard levels for Discharge from Municipal Wastewater Treatment Plant.

[38] Litke D.W. (1999). Review of Phosphorus Control Measures in the United States and Their Effects on Water Quality.

[39] Wu J., Hong Y., Guan F., Wang Y., Tan Y., Yue W., Wu M., Bin L., Wang J., Wen J. (2016). A rapid and high-throughput microplate spectrophotometric method for field measurement of nitrate in seawater and freshwater. Sci. Rep..

[40] Ayala A., Leal L.O., Ferrer L., Cerdà V. (2012). Multiparametric automated system for sulfate, nitrite and nitrate monitoring in drinking water and wastewater based on sequential injection analysis. Microchem. J..

[41] Lin B., Xu J., Lin K., Li M., Lu M. (2018). Low-Cost Automatic Sensor for in Situ Colorimetric Detection of Phosphate and Nitrite in Agricultural Water. ACS Sens..

[42] Kozak J., Latocha K., Kochana J., Wieczorek M., Kościelniak P. (2015). Simultaneous spectrophotometric flow injection determination of phosphate and silicate. Talanta.

[43] Qin J., Li D., Miao Y., Yan G. (2017). Detection of phosphate based on phosphorescence of Mn doped ZnS quantum dots combined with cerium(iii). RSC Adv..

[44] He G., Zhao L., Chen K., Liu Y., Zhu H. (2013). Highly selective and sensitive gold nanoparticle-based colorimetric assay for PO43− in aqueous solution. Talanta.

[45] Suteja Y., Purwiyanto A.I.S. (2018). Nitrate and phosphate from rivers as mitigation of eutrophication in Benoa bay, Bali-Indonesia. IOP Conf. Ser. Earth Environ. Sci..

[46] Isiuku B.O., Enyoh C.E. (2020). Pollution and health risks assessment of nitrate and phosphate concentrations in water bodies in South Eastern, Nigeria. Environ. Adv..

[47] Zhang Z., Lv Y., Zhang W., Zhang Y., Sun C., Marhaba T. (2015). Phosphorus, organic matter and nitrogen distribution characteristics of the surface sediments in Nansi Lake, China. Environ. Earth Sci..

[48] Boonkanon C., Phatthanawiwat K., Wongniramaikul W., Choodum A. (2020). Curcumin nanoparticle doped starch thin film as a green colorimetric sensor for detection of boron. Spectrochim. Acta A Mol. Biomol. Spectrosc..

[49] ICH Expert Working Group (2005). ICH Harmonised Tripartite Guideline: Validation of Analytical Procedure: Text and Methodology.

